# A Rare Clinical Presentation of Multifocal Tuberculous Osteomyelitis of the Right Parietal and Left Calcaneal Bones in an 11‐Year‐Old Male: A Case Report

**DOI:** 10.1155/crdi/4746841

**Published:** 2026-09-26

**Authors:** George Evele, Raoul Ndayong, Gaelle Ngoueffo, Francine Kouya, Ghislain Feudjio, Evans Mbanga, Richard Bardin

**Affiliations:** ^1^ Paediatric Oncology Unit, Mbingo Baptist Hospital, Mbingo, Cameroon, mbingo.org; ^2^ Paediatric Surgery Unit, Mbingo Baptist Hospital, Mbingo, Cameroon, mbingo.org; ^3^ Internal Medicine Unit, Mbingo Baptist Hospital, Mbingo, Cameroon, mbingo.org; ^4^ Radiology Unit, Mbingo Baptist Hospital, Mbingo, Cameroon, mbingo.org; ^5^ Pathology Unit, Mbingo Baptist Hospital, Mbingo, Cameroon, mbingo.org

**Keywords:** case report, osteolytic lesions, paucibacillary, tuberculous osteomyelitis granulomatous inflammation

## Abstract

Paediatric multifocal tuberculous osteomyelitis is a rare and severe clinical entity of extrapulmonary tuberculosis. Globally, *Mycobacterium tuberculosis* (MTB) is the most common cause of necrotising granulomatous inflammation. The diverse clinical presentations of multifocal osteoarticular tuberculosis and its paucibacillary characteristic pose a diagnostic challenge in children. We present the case of an 11‐year‐old male with painless right scalp swelling and painful left foot swelling, associated with intermittent fever. Clinical exam revealed a discharging sinus in the right parietal region, right anterior cervical lymphadenopathy and localised signs of inflammation over the left calcaneus. The C‐reactive protein and erythrocyte sedimentation rate were elevated. The imaging of the skull and left foot showed osteolytic lesions in the right parietal and left calcaneal bones. Cytopathology of the right enlarged cervical lymph node showed necrotising granulomatous inflammation similar to the histopathology report of the left calcaneal lesion biopsy. Although the acid‐fast bacilli smear was negative in the bone biopsy of the left calcaneal lesion, the GeneXpert MTB/RIF Ultra test was positive for MTB. The patient had a good clinical response with surgical intervention and an anti‐tuberculous multidrug regimen. Tuberculosis should be ruled out in children with multifocal osteolytic lesions.

## 1. Introduction

Osteoarticular tuberculosis (TB) is a *Mycobacterium tuberculosis* (MTB) infection involving the bones and joints [1]. It is rare, accounting for about 1%–3% of all tuberculosis cases, and constitutes approximately 10%–20% of extrapulmonary TB diagnoses [1].

Tuberculosis is the leading cause of morbidity and mortality attributed to a single infectious agent worldwide [2]. In 2024, approximately 1.2 million children and adolescents under 15 years old were diagnosed with TB globally (95% uncertainty interval: 0.9–1.5 million) [3]. In the same year, approximately 172,000 children and adolescents under 15 years old died from TB, resulting in an estimated one death every 3 min [3]. Despite advancements in the diagnosis and treatment of TB, approximately 43% of children were misdiagnosed and did not receive care [3]. The Bacille Calmette–Guérin (BCG) vaccine remains the pillar of TB prevention in children [4]. However, as immunity wanes over time, vaccinated individuals become more susceptible to TB infection [5].

Paediatric multifocal osteoarticular tuberculosis is a rare and severe type of extrapulmonary TB in children [6]. It is characterised by the involvement of two or more nonadjacent bone and/or joint lesions, either simultaneously or consecutively, that result from MTB, with or without lung involvement [7]. A tuberculous bone lesion forms when MTB spreads to the bone via blood or lymph [8]. Infected macrophages trigger angiogenesis, aiding the spread of the bacillus [8]. The presence of MTB inside the bone trabeculae stimulates an immune response, leading to the formation of granulomas [8]. As granulomas increase in size, they undergo caseation and liquefactive necrosis, causing bone disintegration and leading to osteolytic or cystic lesions [8, 9]. Early symptoms of TB osteomyelitis are nonspecific, often with pain, swelling, joint stiffness and constitutional symptoms [9]. Osteoarticular TB poses a diagnostic challenge in children due to variability in its clinical presentation and paucibacillary nature, often resulting in higher morbidity and mortality rates [9, 10].

We report a rare case of an 11‐year‐old immunocompetent male with multifocal osteoarticular extrapulmonary TB affecting the right parietal and left calcaneal bones, likely originating from a primary scrofula. This case report has followed the CARE guidelines [11].

## 2. Case Presentation

An 11‐year‐old male was referred to our paediatric surgery clinic from a peripheral health centre in May 2025. He came with a painful left‐sided heel and difficulty bearing weight for 3 months, with no history of trauma. He had a painless swelling on the right side of his scalp for about a month before being referred. Approximately 2 weeks before the referral from the peripheral health centre, the scalp swelling was incised. However, the wound did not heal and continued to discharge bloody fluid despite wound care and empiric antibiotics. The above symptoms were associated with intermittent fever. There was no weight loss or night sweats. He received the Bacille Calmette–Guérin vaccine at birth. He did not have a cough or a history of having been recently exposed to TB. Clinical exam revealed a sinus tract of about 1.0 cm in width over the right parietal region. A solitary right cervical lymph node was about 3 × 3 cm, rubbery mobile and not tender. He had an antalgic gait and a positive ‘heel raise sign’ of the left foot. The left lateral ankle was swollen with no change in colour of the overlying skin. The heel was tender on palpation. The rest of the exam was not contributory.

The blood tests revealed elevated inflammatory markers: C‐reactive protein (CRP) was 163.3 mg/L, and the erythrocyte sedimentation rate (ESR) in the first hour was 118 mm. The complete blood count revealed moderate microcytic hypochromic anaemia. A peripheral blood smear was negative for malignant cells. HIV serology was negative. The basic metabolic panel was normal. Additionally, a cotton swab taken from the scalp wound cultured *Staphylococcus aureus*; however, there was no report of methicillin sensitivity. It was sensitive to fluoroquinolones but resistant to several other antibiotics. Even though a fluoroquinolone was administered, the symptoms continued to worsen.

**FIGURE 1:**
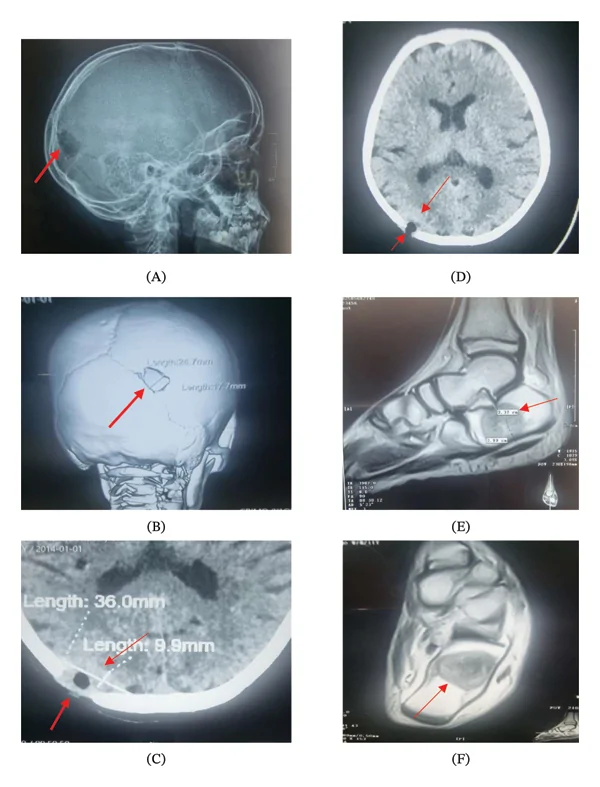
(A) Skull X‐ray (lateral view) osteolytic lesion around the parietal zone. (B) Computed tomography of the skull, destructive bone lesion of the posterior right parietal. (C) CT scan of the head with contrast on parenchyma windows showing right osteolytic parietal lesion with underlying epidural enhancement. (D) CT scan of the head with contrast on parenchyma windows showing right osteolytic parietal lesion with underlying epidural enhancement. (E) T1 sequence showing a hyperintense circular lesion in the left calcaneus. (F) T2/STIR weighted sequence (axial section) showing a hypointense lesion in the left calcaneus.

At the paediatric surgery clinic, an X‐ray of the skull and left foot revealed localised osteolytic lesions of the right parietal bone (Figure 1A) and left calcaneus. The chest radiograph and abdominal ultrasound were normal. Further investigations were requested to characterise these lesions better. A head computed tomography (CT) scan without contrast showed a right parietal osteolytic lesion measuring 27.0 × 8.0 mm and adjacent epidural thickening measuring 36.0 × 10.0 mm (Figure 1B–D). The magnetic resonance imaging (MRI) of the left foot showed an expansive lesion of the left calcaneus, 3 cm in its longest dimension, hyperintense on T1‐weighted images (Figure 1E), hypointense on T2‐weighted images/short tau inversion recovery (STIR) with peripheral enhancement after gadolinium injection, without cortical disruption or extension to adjacent soft tissues (Figure 1F).

A trucut biopsy of the left calcaneal lesion was performed. The histopathology analysis showed a haematolymphoid infiltrative lesion with cells that appeared to be discohesive and monotonous. A clinical suspicion of a bone neoplasm was made. The patient was transferred from the paediatric surgery department to the paediatric oncology unit of the same hospital due to suspected primary bone lymphoma or metastatic bone cancer.

**FIGURE 2:**
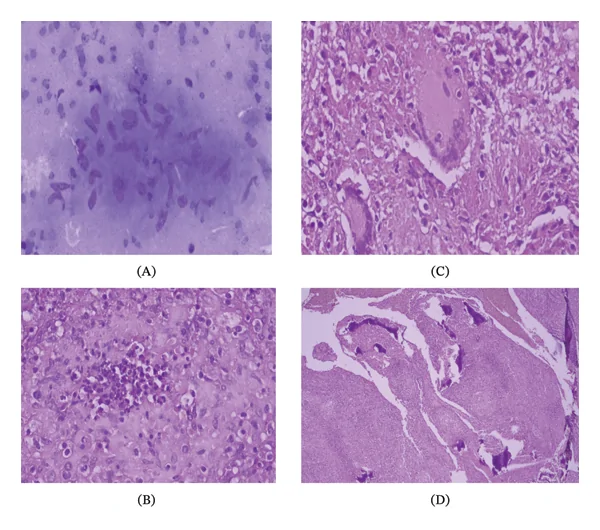
(A) Haematoxylin and eosin (H & E) stain of the aspirated right cervical lymph node, showing granulomatous inflammation. (B) Necrotising granuloma of the left calcaneal lesion. (C) Multinucleate giant cells in the left calcaneal lesion. (D) Necrotic bone in an inflammatory milieu of the left calcaneal lesion.

A Fine Needle Aspiration (FNA) of the right cervical lymph node revealed necrotising granulomatous adenitis, with no malignant cells identified (Figure 2A). Tuberculous lymphadenitis was suspected. A GeneXpert MTB/RIF (rifampicin) test of the lymph node aspirate was requested.

An incision and drainage were performed on the left calcaneal swelling under spinal anaesthesia. Drainage of the purulent content and debridement were done. The collection was sent for antimicrobial culture and antibiotic sensitivity. A biopsy of the calcaneal lesion was taken and sent for histopathological examination and GeneXpert MTB/RIF test. The routine culture did not succeed in isolating any germs, and the standard culture for MTB was not conducted.

The GeneXpert MTB‐RIF Ultra Assay was performed on the lymph node aspirate and calcaneal bone tissue specimens, and low levels of MTB were detected in both specimens, which were sensitive to rifampicin. The tuberculin skin test and the interferon‐gamma release assay were not performed.

Histopathology analysis of the left calcaneus biopsy revealed necrotising granulomatous osteomyelitis (Figure 2B), and malignant cells were not seen. Soft tissue revealed a mixed inflammation, predominantly lymphocytic, with well‐formed granulomas in a necrotic background. The bone had granulomatous inflammation between trabeculae. Granulomas sometimes had a central zone of neutrophils. Langhans‐type giant cells were present (Figure 2C), along with a mixed inflammatory cell population comprising lymphocytes, plasma cells and histiocytes. There were fragments of devitalised bone (Figure 2D), and in non‐necrotic areas, fibrosis was seen. Normal marrow was not present. The Ziehl–Neelsen stain was negative for acid‐fast bacilli, and the Gram stain was negative. No obvious fungal organisms were seen.

The final diagnosis was drug‐susceptible multifocal osteoarticular tuberculosis involving the right parietal and left calcaneal bones associated with a right scrofula. In the absence of a primary pulmonary MTB lesion on chest radiograph, scrofula was assumed to be the primary.

### 2.1. Differential Diagnosis

Multifocal osteoarticular TB can mimic metastatic primary bone cancers in children, such as osteosarcoma and Ewing’s sarcoma [12]. Osteosarcoma is the most common primary bone malignancy in the paediatric population [13]. Osteosarcoma in children can resemble tuberculosis in both clinical and radiological presentations, which can create diagnostic challenges and delays in treatment [14]. Similarly, Ewing’s sarcoma can affect the calcaneus and present with skip and distant metastatic lesions similar to osteoarticular TB [15].

Fungal osteomyelitis shares similarities with TB osteomyelitis in terms of clinical, histological, and radiological features [16, 17]. Clinically, both present with a triad of pain, swelling, and difficulty bearing weight on the affected limb [18]. Histologically, they cause chronic necrotising granulomatous inflammation and contain lipid‐laden macrophages called foam cells [19]. However, necrotising granulomatous inflammation from TB classically results in caseation necrosis, which is rarely seen in fungal osteomyelitis [19]. Radiologically, they show osteolytic lesions [16, 17]. While TB lesions can be localised, diffuse, and sclerotic, those of fungal infections are usually diffuse osteolytic lesions or sclerotic [16, 20]. The most common aetiological agents of fungal osteomyelitis are *Aspergillus* and *Candida* species [17].

Multifocal suppurative osteomyelitis presents with acute symptoms such as fever, bone pain, and swelling of the surrounding tissues [18]. *Staphylococcus aureus* is the most common cause of acute osteomyelitis in children [18]. The clinical presentation of chronic bacterial osteomyelitis can resemble that of osteoarticular tuberculosis both clinically and radiologically [10].

Endemic Burkitt’s lymphoma is the most common childhood cancer in Sub‐Saharan Africa [21]. Advanced stages of Burkitt’s lymphoma can involve the bones and bone marrow, with clinical symptoms similar to osteoarticular TB [9, 21]. Primary Burkitt’s lymphoma of the bone, although rare in children, may present with osteolytic bone lesions resembling those seen in tuberculous osteomyelitis [22].

Rosai‐Dorfman disease (RDD) is a rare non‐Langerhans cell histiocytic disorder that can present with symptoms similar to TB [23]. Classic RDD typically presents with bilateral and painless cervical lymphadenopathy, associated with constitutional symptoms mimicking TB cervical lymphadenitis [23]. Similarly, extranodal bony involvement in Rosai‐Dorfman Disease (RDD) can present as multifocal osteolytic or sclerotic lesions similar to osteoarticular TB [23].

### 2.2. Management

The standard of care was a multidisciplinary team, comprising the paediatric surgery, paediatric oncology, wound care, orthopaedic, internal medicine department, pathology and physiotherapy departments. An anti‐TB multidrug regimen of rifampicin(R), isoniazid(H), ethambutol(E) and pyrazinamide(Z) was initiated immediately. In the intensive phase, he received RHEZ for 2 months and will continue with RH for 10 months. He takes 50 mg of pyridoxine daily to prevent isoniazid‐induced peripheral neuropathy as per routine national guidelines. Finally, he had non‐weight‐bearing physiotherapy care for the left foot for approximately 6 weeks before commencing weight‐bearing exercises. The patient was happy to walk again pain‐free, and the guardian was relieved that cancer concerns were addressed.

### 2.3. Follow‐Up and Outcome

**FIGURE 3:**
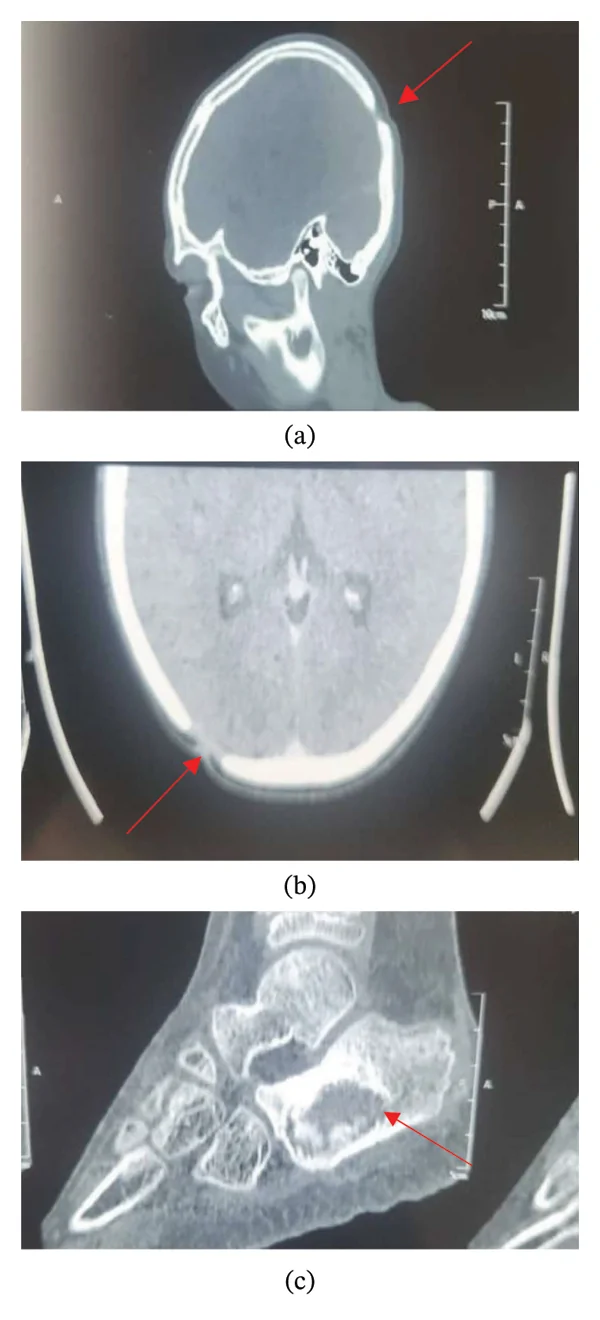
(a) Right parietal osteolytic lesion measuring 27 × 8.0 mm. Disappearance of the epidural thickening adjacent to the known lesion. (b) Right parietal osteolytic lesion measuring 27 × 8.0 mm. Disappearance of the epidural thickening adjacent to the known lesion. (c) CT of the left foot showing osteolytic lesion of the left calcaneus with cortical fracture (sagittal plane).

The wounds over the right scalp and left calcaneus completely healed within the first month of starting the anti‐TB treatment. Subsequently, he has been asymptomatic up to his sixth month of follow‐up. The 6‐month follow‐up CT scans of the head and left calcaneus showed healing of the osteolytic lesions of the right parietal (Figure 3a,b) and left calcaneal lesions (Figure 3c). The liver function tests remain normal, and no significant side effects from the anti‐tuberculous drugs have been noted. Table 1 provides a summary of the time from the onset of symptoms to the 6‐month follow‐up.

**TABLE 1:** Estimated timeline and corresponding presentation and interventions.

Timeline	Clinical presentation/intervention
February 2025	Painful left heel associated with intermittent low‐grade fever.

April 2025	Painless right scalp swelling

April 2025	Scalp swelling incised at a peripheral health centre

May 2025 to August 2025	Consultation with the paediatric surgery and paediatric oncology units. Hospitalised. Incision and drainage of the left heel swelling. Diagnosis of MTB was made, and anti‐TB drugs were commenced. Physiotherapy for 6 weeks. Discharged from the hospital.

November 2025	Evaluated at 6 months with good clinical response and control; imaging requested. To continue the isoniazid and rifampicin regimen for up to 12 months and continue scheduled follow‐up for up to 2 years.

## 3. Discussion

Multifocal tuberculous osteomyelitis is a rare condition, accounting for approximately 5%–15% of osteoarticular TB cases in children [1, 7]. The disease’s rarity can lead to misdiagnosis, as nearly 50% of osteoarticular tuberculosis cases lack radiological evidence of primary pulmonary TB [7]. In children, tuberculous lymphadenitis is the most common form of extrapulmonary TB and can serve as a primary site for seeding to other organs without pulmonary involvement [24]. Additionally, this can occur when the primary Ghon complex in the lungs has been cleared by the patient’s immune system [25]. Cervical tuberculous lymphadenitis (scrofula) accounts for about 60%–90% of cases in TB‐endemic areas [24]. Scrofula can present with unilateral or bilateral lymphadenopathy, affecting one or multiple lymph nodes [24]. The case presented involved multifocal tuberculous osteomyelitis affecting the right parietal bone and the left calcaneus, associated with right tuberculous cervical lymphadenitis. The initial diagnosis for our case was primary bone lymphoma, with differentials of metastatic calcaneal osteosarcoma or Ewing’s sarcoma. Multifocal tuberculous osteomyelitis is often misdiagnosed as metastatic cancer of unknown primary [7]. The key diagnostic clue for TB in our case was the cytopathology of the right cervical lymph node, which showed necrotising granulomatous adenitis—a classic characteristic of TB [8].

Calvarial tuberculosis is a rare occurrence in children [20]. The frontal and parietal bones are particularly affected due to their rich blood supply and MTB’s preference for vascularised spongy bone, which promotes bacterial growth [26]. MTB can spread to the skull bones via blood or lymph from a primary infection, or through penetrating trauma to the skull [10]. Raut et al. [20] studied 42 cases of calvarial tuberculosis and found that most diagnoses were males aged 11–20 years. The majority of patients presented with painless scalp swelling, either with or without a discharging sinus [20]. However, there were rare cases of seizures and meningitis [20]. Our patient was an 11‐year‐old male who presented with painless scalp swelling associated with a non‐healing sinus after incision and drainage. Although *Staphylococcus aureus* was isolated from the wound site, it may have been a secondary infection, as the wound failed to heal despite appropriate antibiotics. Cotton swabs are unreliable for diagnosing MTB or isolating the primary microbe due to potential skin contaminants [27]. Singh and Dutta [28] reported a case of a 10‐year‐old girl with calvarial tuberculosis initially misdiagnosed as a sebaceous cyst. After undergoing surgery, she developed a non‐healing wound along with a discharging sinus [28]. Although calvarial TB is rare in under‐fives, Njock et al. reported on a 4‐year‐old girl with multifocal tuberculous osteomyelitis of the skull and spine associated with pulmonary involvement [29].

Tuberculous osteomyelitis of the foot bones accounts for less than 10% of osteoarticular TB [9]. Tuberculous osteomyelitis of the calcaneus is uncommon [9]. Nonetheless, the calcaneus is the foot bone most commonly affected, likely due to its size and rich vascularisation [9]. The clinical presentation of tuberculous osteomyelitis of the calcaneus is often gradual and nonspecific [9]. Early symptoms may include pain in the lateral or plantar regions of the foot, swelling, joint stiffness, and, later, a discharging sinus may form [9]. When the mechanical pain in the affected foot becomes unbearable, the child may exhibit what is known as the “heel raise sign [30].” Nonspecific symptoms of calcaneal tuberculous osteomyelitis are similar to those of other conditions, such as calcaneal fracture or spur, plantar fasciitis, and bone cancers, which increases the risk of misdiagnosis [9, 10].

The paucibacillary nature of TB in children poses a diagnostic challenge because the low bacterial load is difficult to detect, even with acid‐fast bacilli smears or standard cultures [9]. Additionally, inflammatory markers such as CRP and ESR are not specific indicators for TB [1]. While CRP and ESR can be elevated in TB patients, normal values do not rule out the disease. Similarly, the complete blood count (CBC) in TB cases may show leucocytosis with lymphocyte predominance or fall within normal ranges [9]. Dias et al. [26] reported a case of a 10‐year‐old with calvarial TB where CRP, ESR, and CBC were all normal. Conversely, Chater et al. [9] described elevated inflammatory markers in a 7‐year‐old with TB osteomyelitis of the calcaneus. In our patient, the inflammatory markers were elevated. Imaging modalities such as X‐ray, CT scan, and MRI are crucial in guiding the diagnosis of osteoarticular TB, even though their findings are not specific to TB [20]. Raut et al. [20] reported on three types of tuberculous bone lesions on radiographs in patients with calvarial TB: perforating or localised osteolytic lesions, diffuse or “spreading‐type” osteolytic lesions, and localised sclerotic lesions. Although these radiological findings are not specific to TB, they help raise suspicion of its involvement. However, early signs of osteoarticular TB in children may not be apparent on plain radiographs [9]. CT and MRI play a vital role in guiding early diagnosis of osteoarticular TB in children and in evaluating response to treatment [9, 10]. In all patients with osteoarticular TB, it is recommended to request a plain chest radiograph to exclude pulmonary involvement [7, 10]. Since early bone lesions of osteoarticular TB can be difficult to detect on imaging, a negative radiologic finding does not rule out the disease [7]. Furthermore, bone biopsy is essential for a definitive diagnosis of osteoarticular TB, with specimens sent for histopathological analysis, standard culture, acid‐fast bacilli smear, and molecular testing [10]. The histopathological features of osteoarticular TB include granulomatous inflammation with Langhans giant cells and zones of caseous necrosis [9]. Langhans’ giant cells are a diagnostic hallmark of necrotising granulomatous inflammation, highly suggestive of TB infection [7, 9]. Additionally, standard culture isolates MTB bacilli in fewer than 40% of clinically suspected TB cases [31]. This indicates that over 60% of paediatric TB cases will be diagnosed based on clinical suspicion without microbiological confirmation [32]. Nevertheless, the gold standard for diagnosing MTB is culture using either solid or liquid medium, which can identify the specific phenotype of the MTB complex as well as drug susceptibility [32]. The Ziehl‐Neelsen stain for acid‐fast bacilli is smear‐positive in most osteoarticular TB cases in children [9]. Although standard culture and acid‐fast bacilli smears are important for diagnosing osteoarticular TB in children, negative results do not exclude MTB infection [10]. The World Health Organisation (WHO) recommends rapid molecular testing for MTB DNA using the GeneXpert MTB/RIF Ultra or Truenat assays for a definitive diagnosis of extrapulmonary TB, over culture and acid‐fast smears [33]. The GeneXpert MTB/RIF Ultra not only detects MTB DNA but also tests for rifampicin resistance, a crucial prognostic marker [10, 33]. Sun et al. [34] demonstrated in their study that the GeneXpert MTB/RIF Ultra assay exhibits superior sensitivity compared to both the GeneXpert MTB/RIF and conventional culture methods in the diagnosis of osteoarticular tuberculosis. Routine indirect testing of MTB infection with both tuberculin skin test and interferon‐gamma release assays is not recommended except in certain clinically suspected cases with initially negative investigations [25]. In children, TB infection is usually recent, while in adults, it often stems from reactivation of a past asymptomatic infection [10, 20]. Multimodality testing in children with clinically suspected multifocal osteoarticular TB increases the diagnostic accuracy [10].

The multidisciplinary approach is the gold standard for managing paediatric multifocal osteoarticular TB [10]. Treatment mainly involves anti‐tuberculous multidrug therapy, with or without surgery [4, 10]. Although the WHO recommends a 12‐month duration of anti‐TB therapy (ATT), the optimal duration is debated [9, 10]. The intensive phase uses four drugs: rifampin (10 mg/kg), isoniazid (5 mg/kg), ethambutol (15 mg/kg) and pyrazinamide (25 mg/kg) [10]. Variability exists in the continuation phase across guidelines, with differences in drug regimen and duration [10]. Pyridoxine supplementation is administered to prevent isoniazid‐induced peripheral neuropathy, especially in patients with malnutrition or HIV [35]. Some local protocols recommend pyridoxine supplementation for all patients on isoniazid regimens, while others do not, especially if the patient is healthy and can obtain the daily dietary requirement from food [35]. Our patient received pyridoxine supplementation according to the national protocol, although there was no clear indication for it. The patient completed the intensive phase and will receive RH for 10 months. Surgical interventions depend on the disease site and severity, including incision and drainage, debridement and grafting [10]. Generally, drug‐susceptible osteoarticular TB has a good prognosis [10, 26]. Regular clinical monitoring up to 2 years is the key, as complications such as drug resistance, relapse or severe adverse effects of the anti‐TB drugs may occur and require immediate intervention [7, 10].

## 4. Conclusion

Multifocal osteoarticular tuberculosis in children is a rare condition that can be misdiagnosed as cancer. A succinct clinical examination is the key to diagnosing multifocal osteoarticular TB. Multimodal tests enhance the diagnostic accuracy of multifocal osteoarticular tuberculosis in children due to its low bacterial load and symptoms that often resemble malignancy. The outcomes are generally good with the standard multidrug ATT.

### 4.1. Limitations of the Case Report

While the nucleic acid amplification test is recommended for the rapid diagnosis of MTB, it is not considered the gold standard because it cannot differentiate between the various species of the MTB complex. Conventional culture for MTB was not conducted in this case, despite being recommended for all suspected MTB cases to enhance diagnostic clarity. The absence of culture confirmation is a limitation of the study, and access to mycobacterial culture should complement the molecular diagnostic assays whenever feasible. Routine culture of the scalp wound swab was performed, but it offers limited diagnostic value due to contamination from skin microbes. The methicillin sensitivity of the isolated *Staphylococcus aureus* from the scalp wound was not tested; although it showed sensitivity to fluoroquinolones, the overall response was poor. Healthy patients with TB do not require routine pyridoxine supplementation to prevent isoniazid‐induced peripheral neuropathy, as the amount obtained from a daily balanced diet is sufficient. High doses of pyridoxine may also reduce the antibacterial effect of isoniazid.

### 4.2. Strengths of the Case Report

A multimodal testing method was used along with tissue analysis of specimens from the affected sites. The characteristic sign of granulomatous inflammation with caseous necrosis points to MTB, especially in endemic areas. The GeneXpert MTB/RIF Ultra assay provided rapid results, allowing for the immediate initiation of an anti‐TB regimen, which prevented further bone destruction and reduced the risk of tuberculous meningitis. A multidisciplinary team helps reduce diagnostic delays and misdiagnoses, ultimately improving patient outcomes.

## Author Contributions

All authors have contributed to this case report.

George Evele: conceptualisation, data curation, investigation, methodology, supervision, visualisation and writing –original draft and review and editing. Raoul Ndayong: methodology, investigation and review and editing. Gaelle Ngoueffo: methodology and writing–original draft and review and editing. Ghislain Feudjio: investigation and review and editing. Evans Mbanga: investigation and review and editing. Francine Kouya: methodology and review and editing. Richard Bardin: investigation, validation, supervision and review and editing.

## Funding

No funding was obtained for this case report.

## Disclosure

A preprint of this article has previously been published (Evele et al. [36]). All authors read and approved the final manuscript.

## Ethics Statement

The ethical clearance for this case report was obtained from the Cameroon Baptist Convention Health Board Institutional Review Board, holding an IRB study number of IRB2025‐120. The date of approval was January 23, 2026.

## Consent

The patient’s mother signed the written informed consent form for this case report and images for this publication.

## Conflicts of Interest

The authors declare no conflicts of interest.

## Supporting Information

Additional supporting information can be found online in the Supporting Information section.

## Supporting information


**Supporting Information** CARE‐checklist‐English‐2013 (1)

## Data Availability

The data that support the findings of this study are available from the corresponding author upon reasonable request.
