# Supplementary material for: A Rare Clinical Presentation of Multifocal Tuberculous Osteomyelitis of the Right Parietal and Left Calcaneal Bones in an 11‐Year‐Old Male: A Case Report
Source: Case Rep Infect Dis. 2026 Sep 26;2026:4746841. doi: 10.1155/crdi/4746841 (PMC13615510; doi:10.1155/crdi/4746841)
Supplement: Supplementary file 1 [file CRDI-2026-4746841-s001.docx]

# CARE Checklist of information to include when writing a case report

**Topic	Item	Checklist item description	Reported on Line**

**Title	1**	The diagnosis or intervention of primary focus followed by the words “case report” . . . . . . . . . . . . . . . . . .	Yes

**Key Words	2**	2 to 5 key words that identify diagnoses or interventions in this case report, including "case report"	Yes

**Abstract**

**(no references)**

**3a**	Introduction: What is unique about this case and what does it add to the scientific literature?	Yes

**3b**	Main symptoms and/or important clinical findings . . . . . . . . . . . . . . . . . . . . . . . . . . . . . . . . . . . . . . . . . . . . . . . . . . .	Yes

**3c**	The main diagnoses, therapeutic interventions, and outcomes	Yes

**3d**	Conclusion—What is the main “take-away” lesson(s) from this case?	Yes

**Introduction	4**	One or two paragraphs summarizing why this case is unique (**may include** reference**s**)	Yes

**Patient Information	5a**	De-identified patient specific information	Yes

**5b**	Primary concerns and symptoms of the patient	Yes

**5c**	Medical, family, and psycho-social history including relevant genetic information	Yes

**5d**	Relevant past interventions with outcomes	Yes

**Clinical Findings**

**Timeline**

**Diagnostic Assessment**

**Therapeutic Intervention**

**Follow-up and Outcomes**

6 Describe significant physical examination (PE) and important clinical findings	Yes
7 Historical and current information from this episode of care organized as a timeline	Yes

**8a**	Diagnostic testing (such as PE, laboratory testing, imaging, surveys).	Yes

**8b**	Diagnostic challenges (such as access to testing, financial, or cultural)	Yes

**8c**	Diagnosis (including other diagnoses considered)	Yes

**8d**	Prognosis (such as staging in oncology) where applicable	Yes

**9a**	Types of therapeutic intervention (such as pharmacologic, surgical, preventive, self-care) . . . . . . . . . . . . . . . . .	Yes

**9b**	Administration of therapeutic intervention (such as dosage, strength, duration)	Yes

**9c**	Changes in therapeutic intervention (with rationale)	Yes

**10a**	Clinician and patient-assessed outcomes (if available)	Yes

**10b**	Important follow-up diagnostic and other test results	Yes

**10c**	Intervention adherence and tolerability (How was this assessed?)	Yes

**10d**	Adverse and unanticipated events	Yes

**Discussion	11a**	A scientific discussion of the strengths AND limitations associated with this case report	Yes

**11b**	Discussion of the relevant medical literature **with references**	Yes

**11c**	The scientific rationale for any conclusions (including assessment of possible causes)	Yes

**11d**	The primary “take-away” lessons of this case report (without references) in a one paragraph conclusion	Yes

**Patient Perspective	12**	The patient should share their perspective in one to two paragraphs on the treatment(s) they received . . . .	Yes

**Informed Consent	13**	Did the patient give informed consent? Please provide if requested . . . . . . . . . . . . . . . . . . . . . . . . . . . . . . . . . . . . . .YES	**Yes** **No**
